# Supplementary material for: A combination of chitooligosaccharide and lipochitooligosaccharide recognition promotes arbuscular mycorrhizal associations in Medicago truncatula
Source: Nat Commun. 2019 Nov 6;10:5047. doi: 10.1038/s41467-019-12999-5 (PMC6834629; doi:10.1038/s41467-019-12999-5)
Supplement: Supplementary file 1 — Supplementary Information [file 41467_2019_12999_MOESM1_ESM.pdf]

Supplementary figures

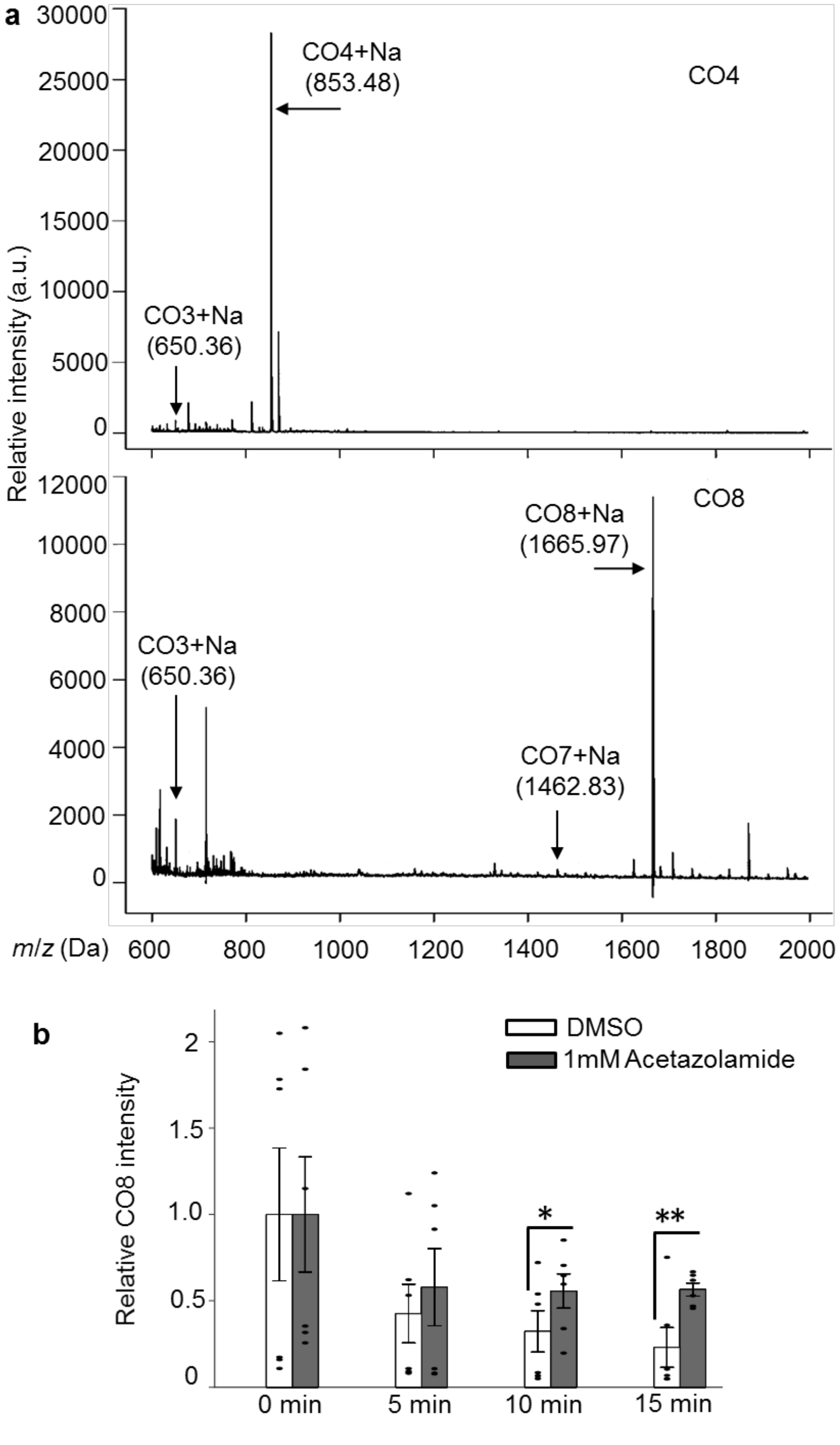

Supplementary Figure 1. CO samples and their degradation in *M. truncatula* roots. a Representative MALDI-TOF MS spectra of the CO4 (upper panel) and CO8 (lower panel) with relevant molecular weights indicated. Note: all CO molecules exist in the form of sodium salts resulting from their purification. The individual molecular weights shown are the sum mass of COs and Na (23 Da). **b** Quantification of CO8 incubated with *M. truncatula* lateral roots in the presence of either DMSO or Acetazolamide. The zero minute sample was used as a reference. This experiment was repeated three times with similar results. Significance: $^*P<0.05$ and $^{**}P<0.01$ measured by the Student's *t*-test.

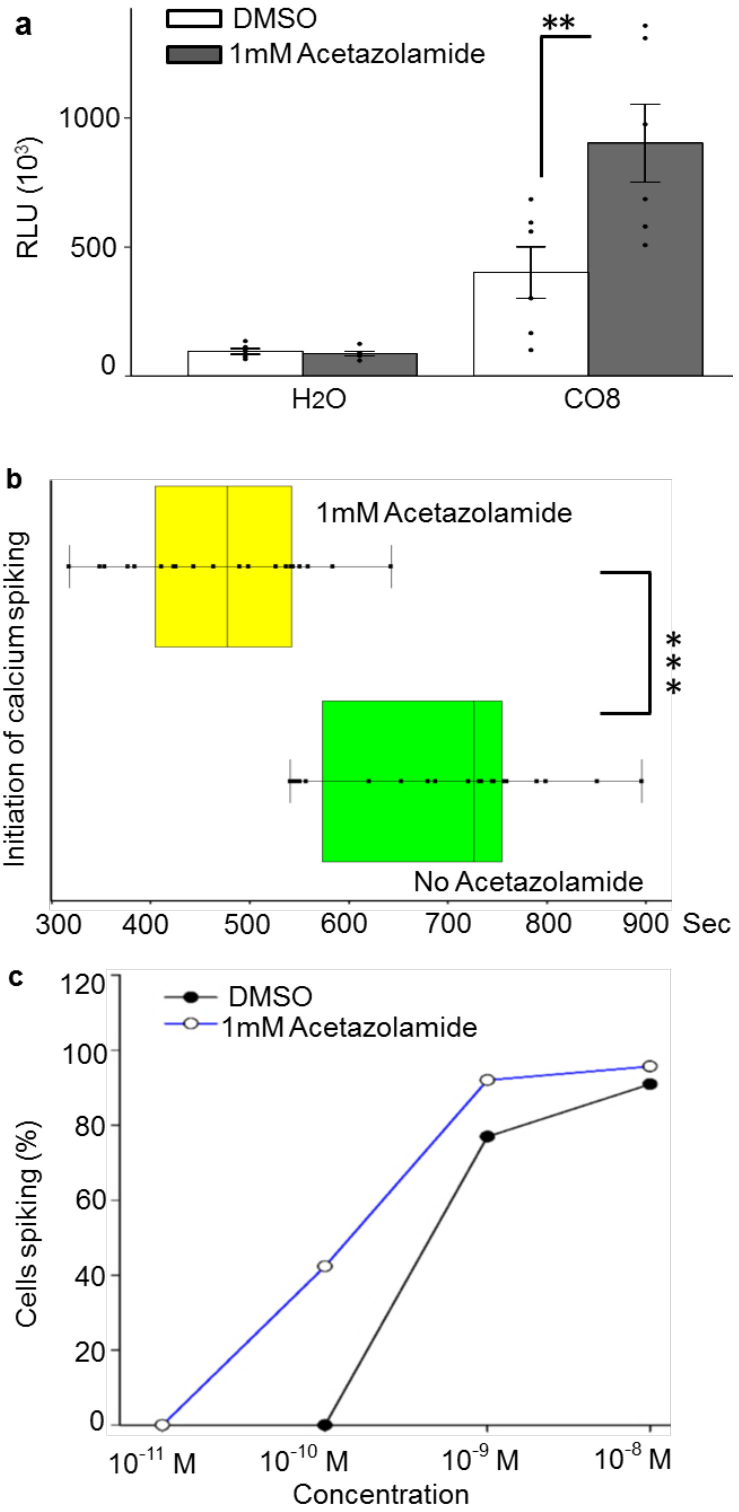

Supplementary Figure 2. The chitinase inhibitor Acetozolamide enhances CO8 responses. **a** ROS production in roots of *M. truncatula* activated by 10^{-9} M CO8 with or without Acetazolamide at indicated time points (n=6, mean ±s.e.m. $P<0.01$ by Student's *t*-test). This experiment was repeated three times with similar results. **b** Atrichoblasts on lateral roots of *M. truncatula* wild type were measured either for the time until initiation of calcium oscillations following 10^{-8} M CO8 treatment or **c** the percentage of cells responding with calcium oscillations. Note that the addition of Acetazolamide reduces the timing and enhances the number of responsive cells. *** Significant difference, $P<0.001$ by Student's *t*-test.

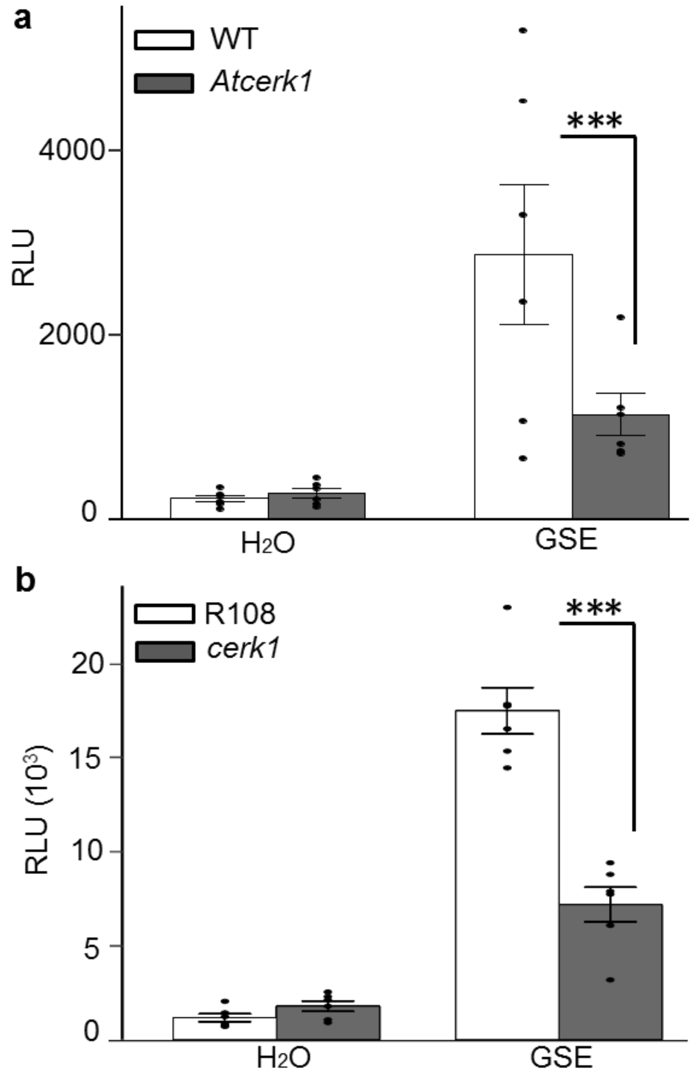

Supplementary Figure 3. ***R. irregularis*** **germinated spore exudates (GSE) induce ROS in** ***Arabidopsis*** **and** ***Medicago*** **dependent on** ***CERK1***. Measurement of reactive oxygen species after treatment with 10 times concentrated GSE in the leaf discs of *A. thaliana Col-0* and *cerk1* **(a)** and roots of *M. truncatula* wild type R108 and *Mtcerk1* **(b)**. Shown are the average values ± standard errors of relative luminescent units (RLU) (n=6). ***significant difference relative to WT, $P<0.001$, Student's *t*-test. These experiments were repeated twice with similar results.

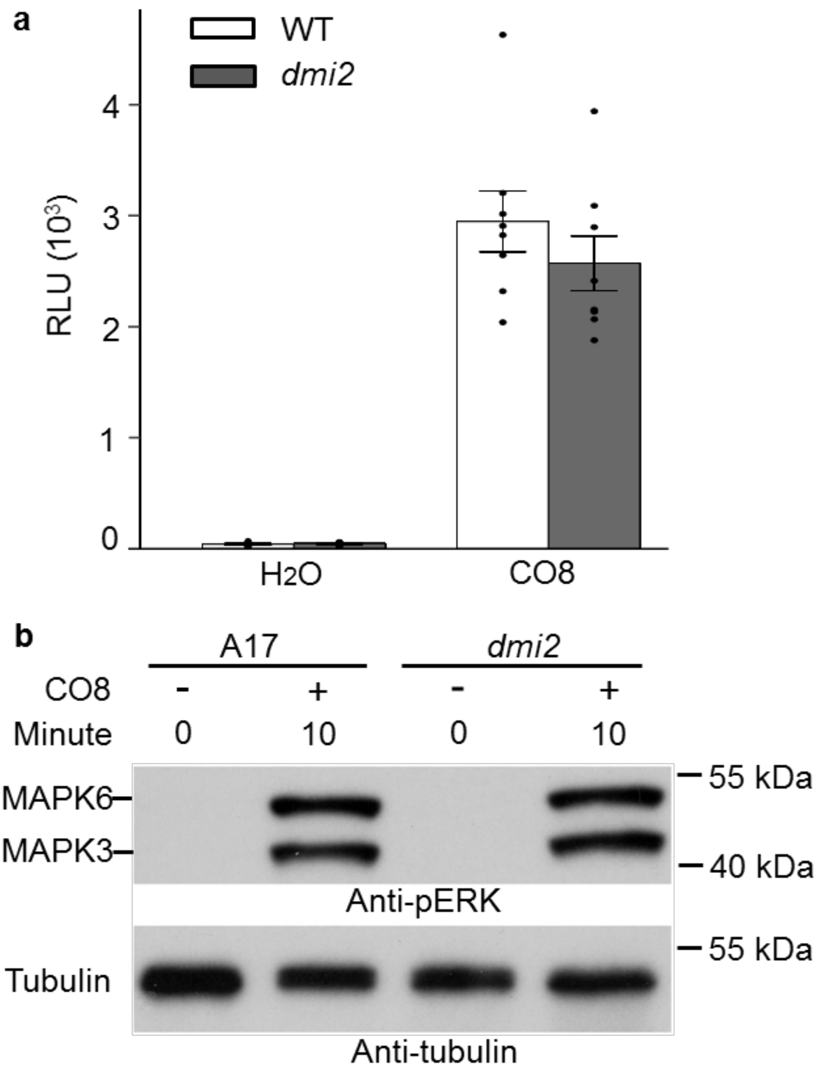

Supplementary Figure 4. DMI2 is not required for CO8-induced defense responses. a ROS production in roots of WT and *dmi2* treated with 10^{-6} M CO8 (n=8, mean ±s.e.m.). This experiment was repeated three times with similar results**. b** Activation of MAPK3 and MAPK6 after treatment of 10^{-6} M CO8 at 10 min in *M. truncatula* wild type and *dmi2.*

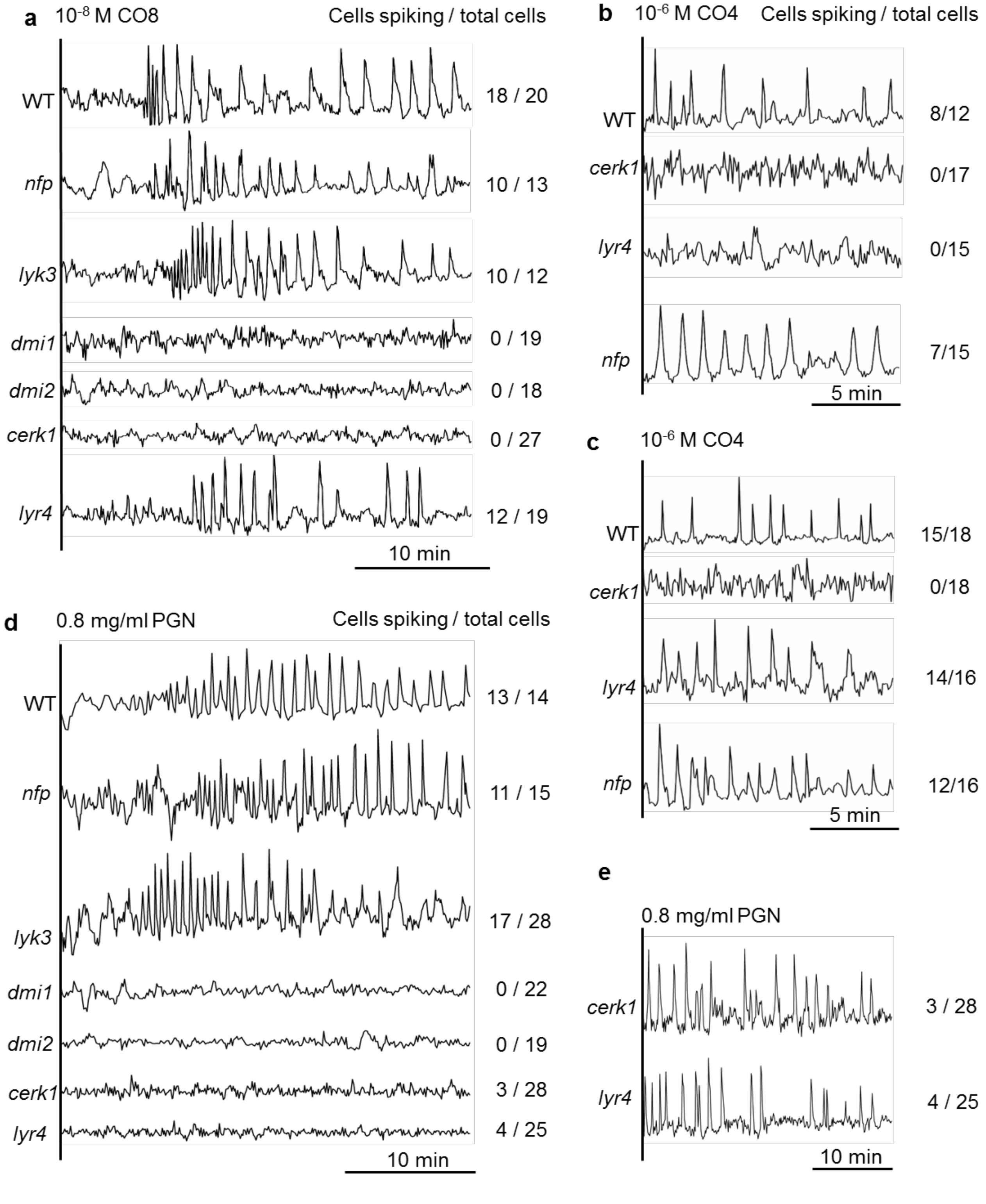

Supplementary Figure 5. The genetic dependencies of CO8, PGN and CO4 induced calcium oscillations in *M. truncatula* lateral roots. *M. truncatula* wild type and mutant lateral roots were treated with 10^{-8} M CO8 **(a)**, 10^{-6} M CO4 **(b, c)** or 0.8 mg/ml PGN **(d, e)** and calcium responses measured in both trichoblasts **(b)** and atrichoblasts **(a, c, d, e).** Representative traces for the majority responses are shown. The majority of atrichoblasts in *cerk1* and *lyr4* show no response to PGN (**d**), however, there are a few responsive cells (**e**).

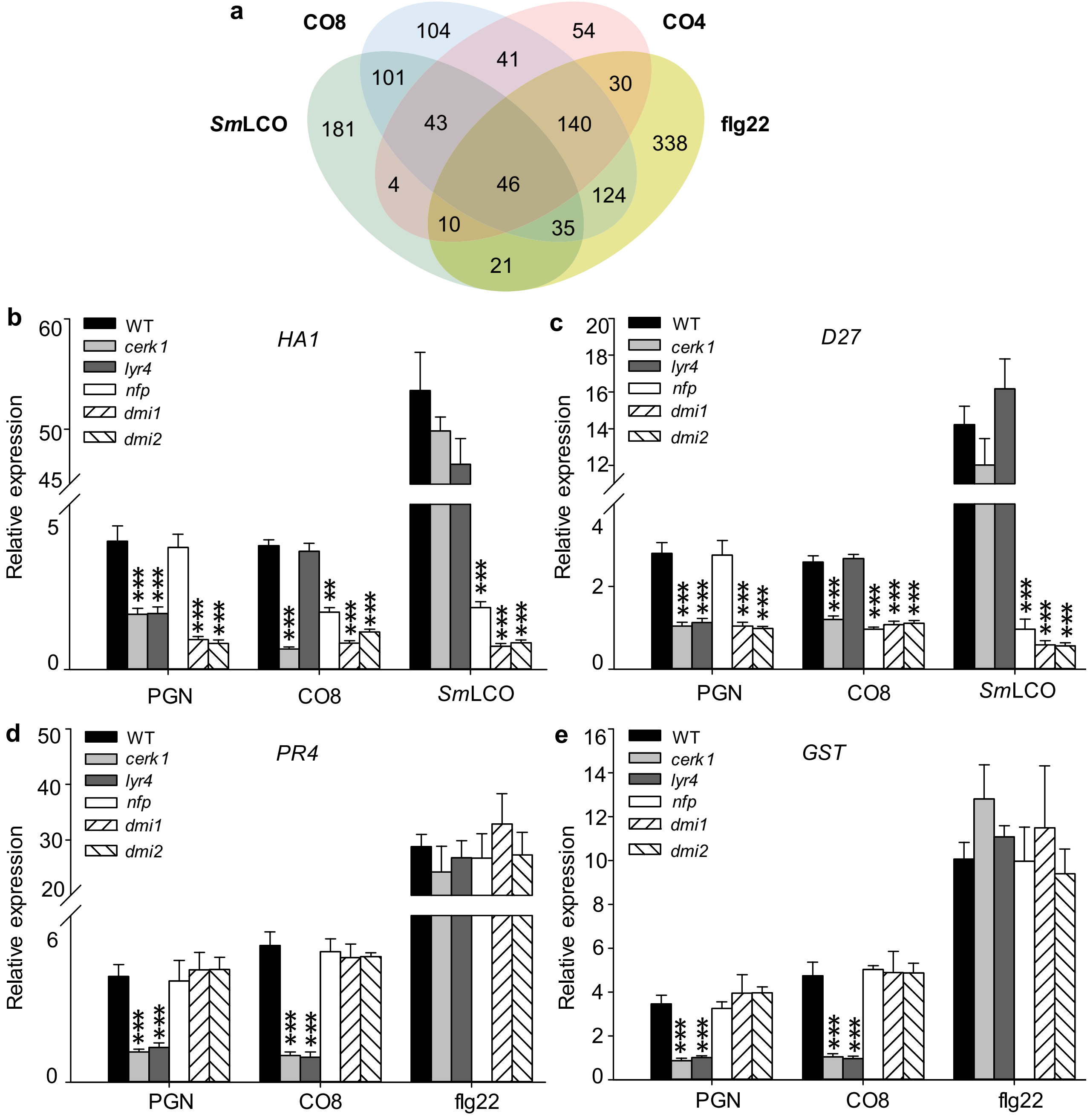

Supplementary Figure 6. CO8 and PGN-induced symbiotic and defense-related genes expression in *M. truncatula* roots. a The distribution of genes induced by CO8, CO4, flg22 and *Sm*LCO in WT plants during RNAseq analysis. qRT-PCR validation of representative symbiotic genes *HA1* and *D27* (**b**,**c**) and defense-related genes *PR4* and *GST* (**d**,**e**) in responding to 0.4 mg/ml PGN, 10^{-7} M CO8, 10^{-8} M *Sm*LCO and 10^{-7} M flg22, respectively. The induction of these genes was detected in wild type and mutants. Relative fold changes compared to individual DMSO treatments are shown. (mean ±s.e.m.; n=3; ***significant difference relative to wild type treatment by Student *t*-test, $P<0.001$; ** $P<0.05$). These experiments were repeated three times with similar results.

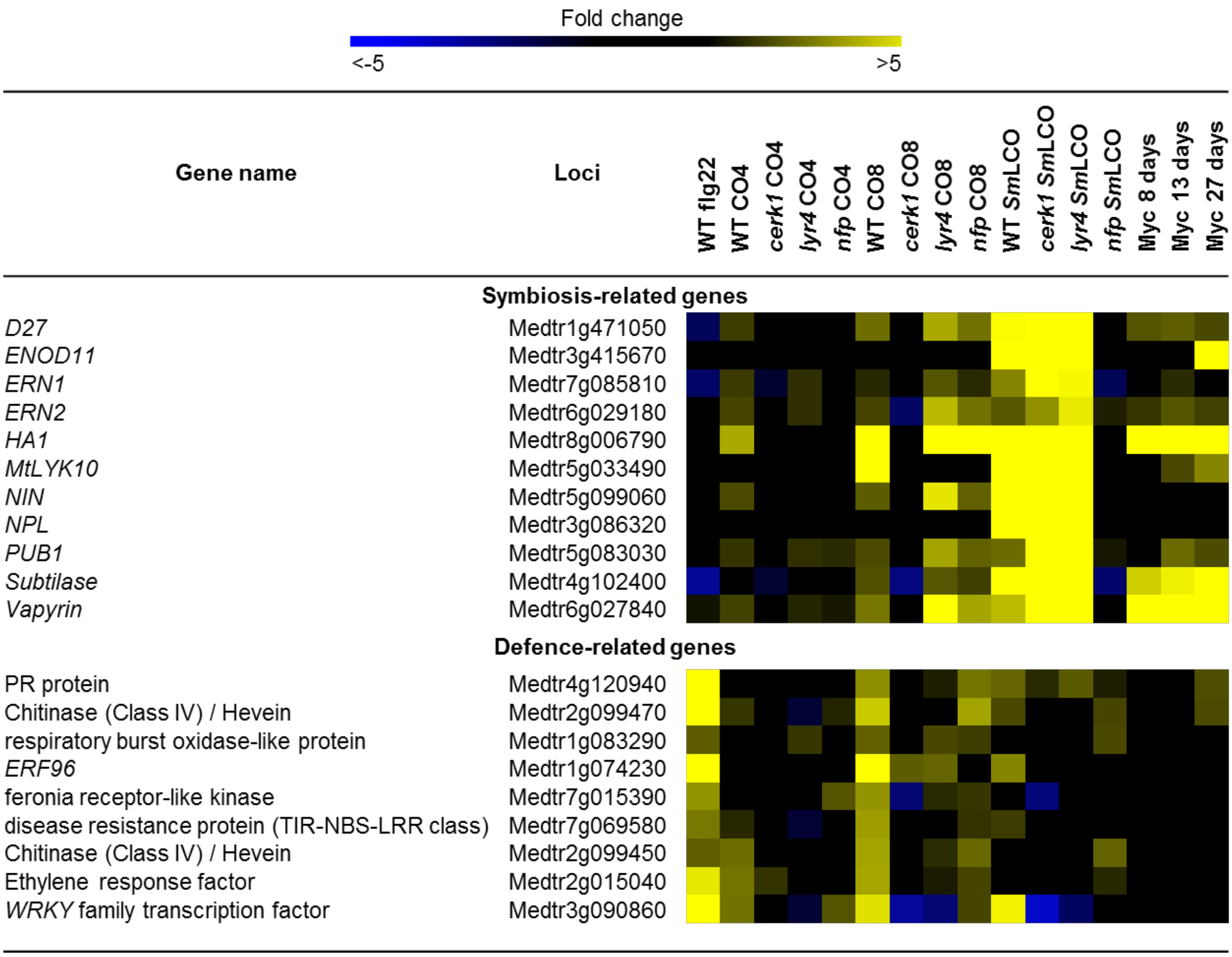

Supplementary Figure 7. Differential expression of representative symbiotic and defense-related genes in wild type and mutants of *M. truncatula* roots responding to flg22, CO4, CO8, *Sm*LCO and mycorrhizal fungi.

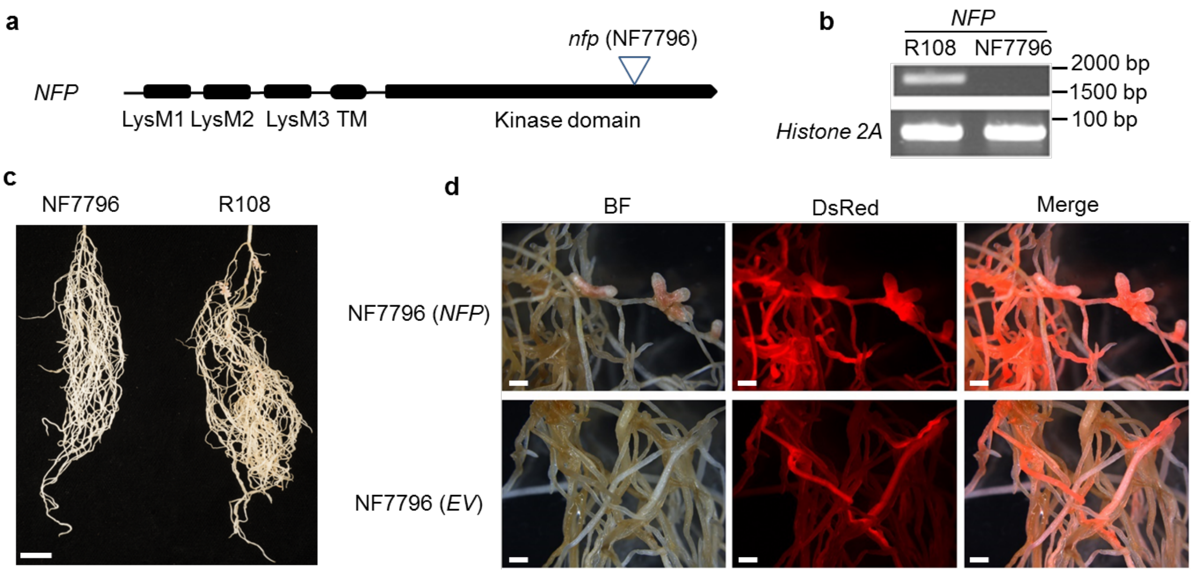

Supplementary Figure 8. Characterisation of a new *nfp* allele in the R108 background. **a** The structure of *NFP* showing the position of the *Tnt1* insertion in the mutant allele. Predicted LysM domains, transmembrane domains (TM) and kinase domains are indicated. **b** Semiquantitative RT-PCR to detect the transcript levels of *NFP* in *M. truncatula* wild type and mutant roots. *Histone 2A* is used as a loading control. **c** NF7796 is defective for nodulation. The picture was taken of 3-week-old plants grown in soil with *S. meliloti* 1021. **d** Expression of *NFP* can complement the nodulation phenotype of NF7796 mutant. NF7796 roots were transformed with empty vector (EV) or *NFP* genomic sequence driven by its native promoter. DsRed was present in the plasmids to act as a transformation marker. Images were taken 4 weeks post inoculation with *S. meliloti* 1021. The pictures show a fluorescence image corresponding to the bright field (BF) image. Scale bars=1 cm **(c)** and 1 mm **(d)**.

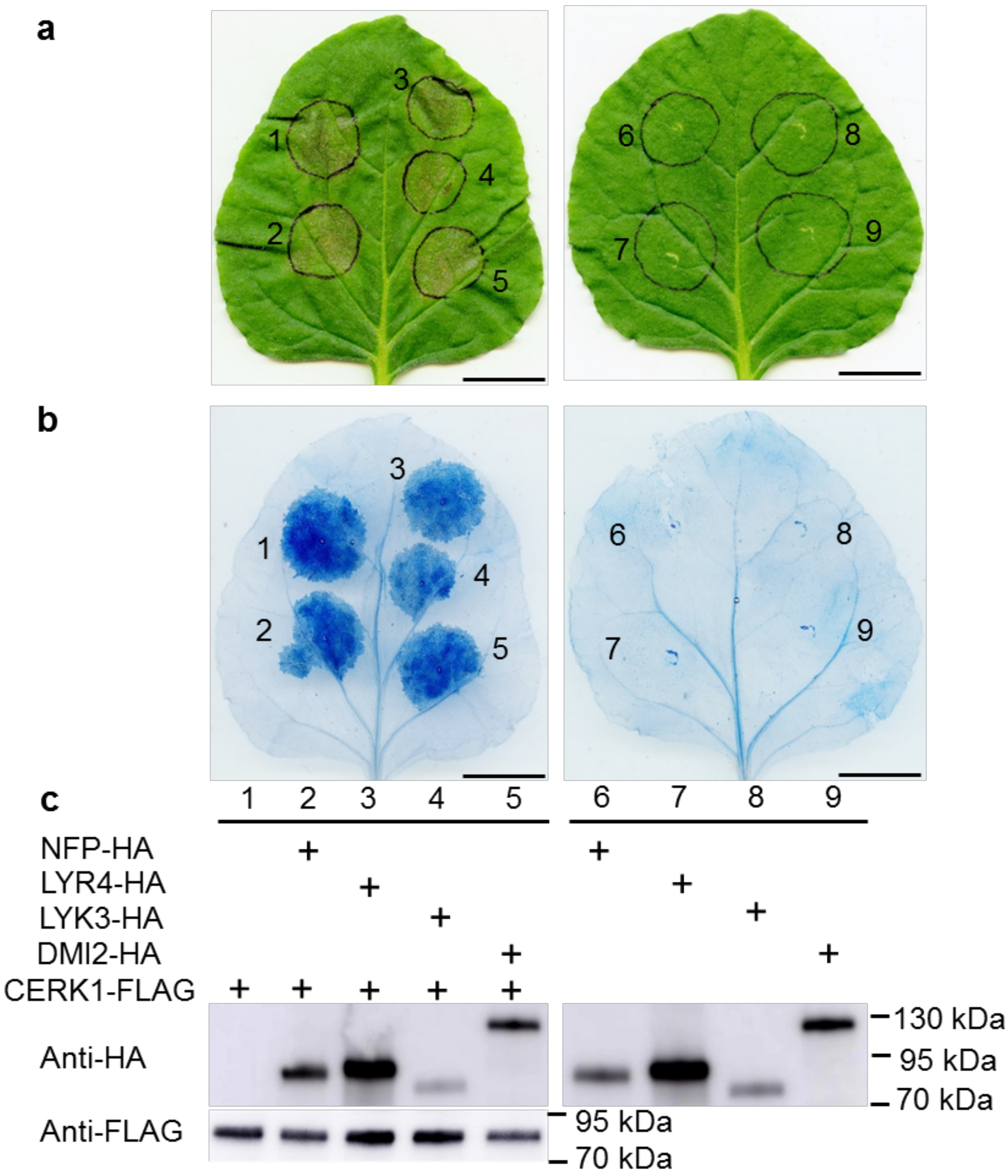

Supplementary Figure 9. Overexpression of NFP has no effect on *Mt*CERK1 induced cell death. a Transient expression of *MtCERK1* in *N. benthamiana* leaves induces cell death whether expressed alone (1) or in combination with *NFP* (2), *LYR4* (3), *LYK3* (4) or *DMI2* (5). **b** Individual expression of *NFP* (6), *LYR4* (7), *LYK3* (8) or *DMI2* (9) has no effect. **c** Expression levels of receptor measured by western blotting with anti-HA and anti-FLAG antibodies. (Scale bars=2 cm).

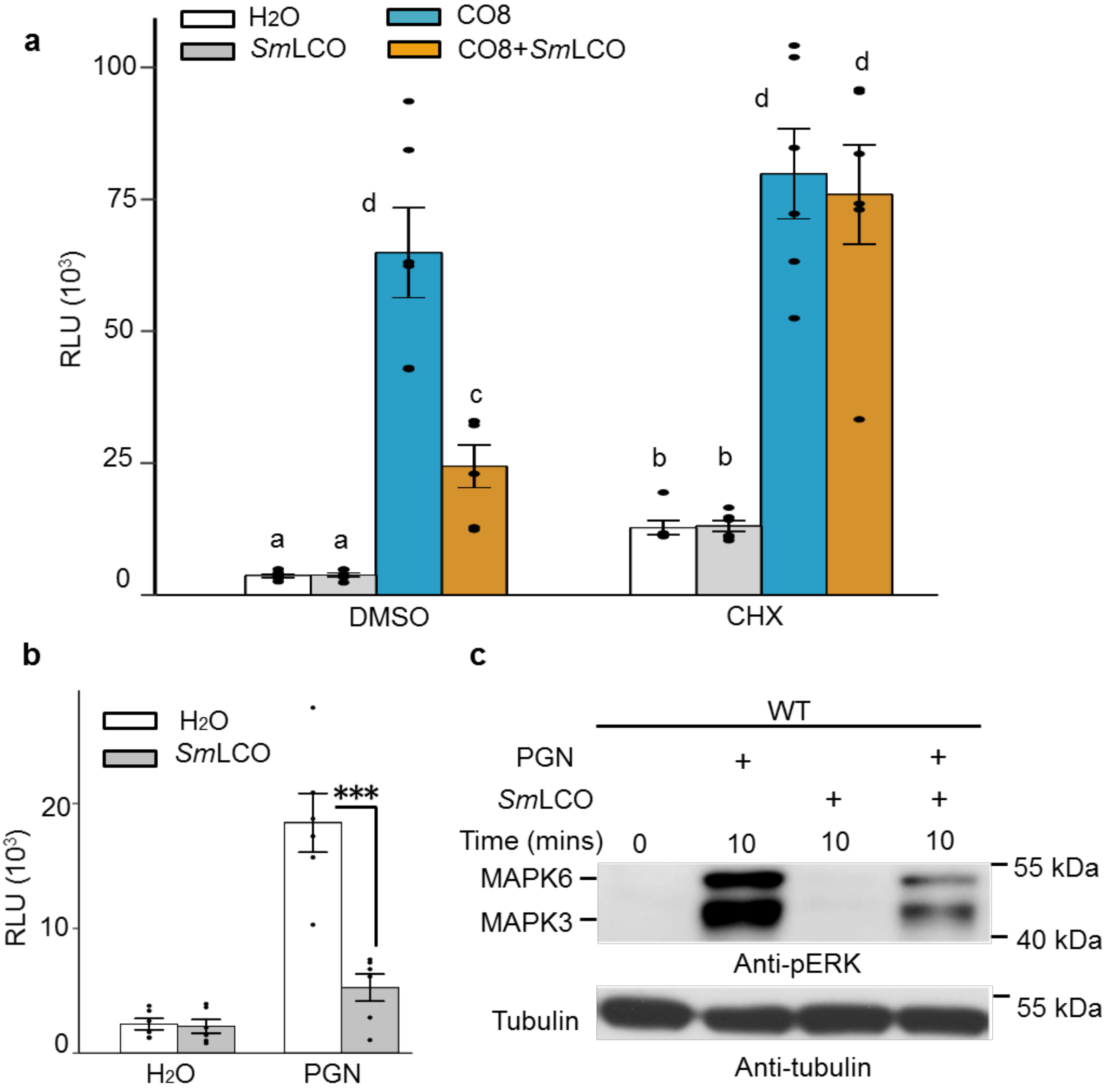

Supplementary Figure 10. *Sm*LCO suppression of immunity signaling occurs with PGN and CO8 elicitation and is inhibited by cycloheximide (CHX). a *M. truncatula* roots were pre-incubated with the protein synthesis inhibitor 100 µM cycloheximide for 4 hours before *Sm*LCO treatment. This experiment was repeated three times with similar results. Letters denote statistically significant groupings calculated with Mann-Whitney Rank Sum Test (n=6, mean ±s.e.m.). ROS production **(a,b)** and MAPK3/6 activation **(c)** in *M. truncatula* wild type roots pre-treated with 10^{-7} M *Sm*LCO for 30 minutes and then incubated with the same amount of PGN/CO8 or a mixture of PGN/CO8 with *Sm*LCO. ***denotes significant difference relative to PGN treatment alone, $P<0.001$, Student's *t*-test. RLU: relative luminescent units. The phosphorylated bands of MAPK3/6 as shown by immunoblot analysis using an anti-pERK antibody.

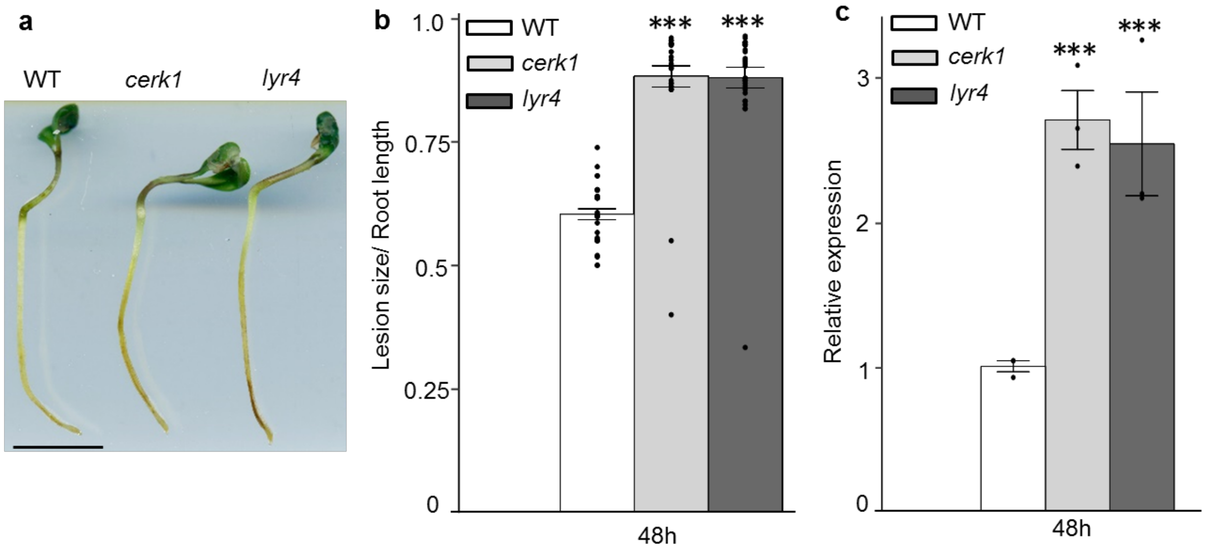

Supplementary Figure 11. *MtCERK1* and *LYR4* are involved in *Phytophthora palmivora* colonization. Wild type, *Mtcerk1* and *lyr4* seedling roots were inoculated with *P. palmivora* spores to record disease symptoms **(a)**, quantify lesion size **(b)** and quantify pathogen levels **(c)**. (n=30 for **b** and n=3 for **c**, mean ±s.e.m.). *** denotes significant difference relative to wild type plants, $P<0.001$, Student's *t*-test. (Scale bars=1 cm). These experiments were repeated twice with similar results.

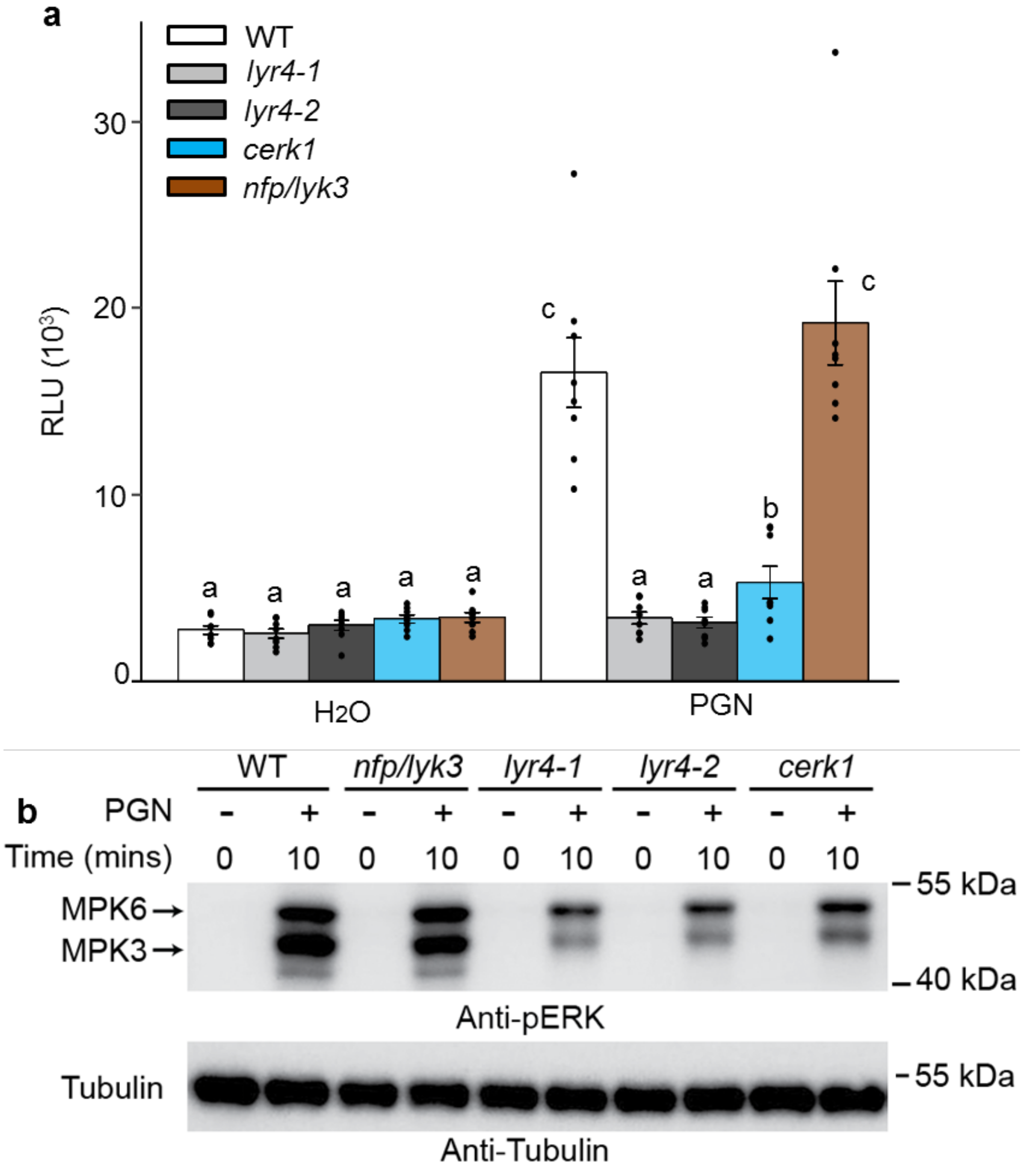

Supplementary Figure 12. LYR4 and *Mt*CERK1 are required for PGN-induced immunity in *M. truncatula* roots. a The relative luminescent units for ROS production were measured in the roots of *M. truncatula* wild type and different receptor mutants after treatment of 0.4 mg/ml PGN. Letters denote statistically significant groupings calculated with Mann-Whitney Rank Sum Test (mean ±s.e.m., n=8; $P < 0.01$). This experiment was repeated three times with similar results. **b** *M. truncatula* wild type and receptor mutants were incubated with 0.4 mg/ml PGN for 10 minutes and the phosphorylation of MAPK3 and MAPK6 was detected by anti-pERK antibody. A duplicate blot was used to detect tubulin protein levels using an anti-tubulin antibody.

Supplementary Table 1. Nuclear calcium oscillations induced by chitin oligomers in atrichoblasts of *M. truncatula* lateral roots

Chitin oligomers (10^{-8} M)	Cells spiking / total cells
CO8	20 / 22
CO7	16 / 19
CO6	13 / 17
CO5	20 / 29
CO4	17 / 24
CO3	0 / 30
CO2	0 / 34

Numbers denote cells responding relative to total cells analysed.

Supplementary Table 2. Acetazolamide does not induce nuclear calcium oscillations

1mM Acetazolamide	Cells spiking / total cells
Trichoblast	0 / 16
Atrichoblast	0 / 17

Numbers denote cells responding relative to total cells analysed.

Supplementary Table 3. Calcium responses in complemented *lyr4* and *Mtcerk1* roots

	10^{-8} M CO8<br>Cells spiking / total cells	
cerk1 (Empty vector)	0 / 18	Atrichoblast
cerk1 (*CERK1*)	14 / 15	
cerk1 (Empty vector)	0 / 21	Trichoblast
cerk1 (*CERK1*)	6 / 8	
lyr4 (Empty vector)	0 / 9	Trichoblast
lyr4 (*LYR4*)	8 / 10	

Numbers denote cells responding relative to total cells analyzed.

Supplementary Table 4. The primers used in this study

Primers name	Sequence (FW 5'-3')	Use
LYK10-F	AGAAGCTACGAGCCAAGGTAGC	qPCR *LYK10*
LYK10-R	AGGTAGCCTGGTGTTCCAACAAG	
PR10-F	GGCTCAAATGGAGGGTCTATTG	qPCR *PR10*
PR10-R	GCTTTGCCTTCCTCAACCT	
Chitinase-F	GGCTGACATCCTTACACAAGA	qPCR *Chitinase*
Chitinase-R	AGAATTGAGGGCATCGAGAAA	
HA1-F	CCATGATAGCGCAGAAACAATC	qPCR *HA1*
HA1-R	CCTTCCCGTTTCCTTTCCTATT	
Vapyrin-F	GCCAGTTGCATTTAGGATTCA	qPCR *Vapyrin*
Vapyrin-R	GCACCTGGAGCAAGAACACT	
Histone 2A-F	CTTTGCTTGGTGCTGTTTAGATGG	qPCR *Histone 2A*
Histone 2A-R	ATTCCAAAGGCGGCTGCATA	
NFP-F	ATGTCTGCCTTCTTTCTTCCTTC	NF7796 genotyping
NFP-R	ACGAGCTATTACAGAAGTAACAAC	
D27-F	AGTTCTTGCAAGGCCTACAGATG	qPCR *D27*
D27-R	TGATTCCTGTTGCTGCTTGAACAC	
GST-F	GGACCCTTACAAACGATCACA	qPCR *GST*
GST-R	TTCCTGTCCACACCTTCTTTC	
PR4-F	CTTGCGGCAAATGCTTGACTGTG	qPCR *PR4*
PR4-R	TGAACGCCCTGTCCATTGGTATC	
PpEF1a-F	CAAGATCCCGTTCGTGCCTA	qPCR *Phytophthora palmivora EF1a*
PpEF1a-R	GCGTTCAGGTTGTCAAGAGC	
NFP-F1	TCGAGGTACCATGTCTGCCTTCTTTCTTCCTTC	NFP-HA sequencing
NFP-R1	CTACATCGATACGAGCTATTACAGAAGTAACAAC	
NFP-F2	TGTTAGCTGAAAACAATCATAAC	
LYR4-F	TCGAGGTACCATGGCATGGCAAACCTTAACAAC	LYR4-HA sequencing
LYR4-R	CAACGTCGACTCTACTATCAGAAACTTGGCTAAC	
LYR4-F1	AATGAAGCTAATGAACTTTCTTC	
LYK3-F	TCGAGGTACCATGAATCTCAAAAATGGATTACTA	LYK3-HA sequencing
LYK3-R	CAACCTCGAGTCTAGTTGACAACAGATTTATGAG	
LYK3-F1	AGCATAGGAAGTGGGATAGTGTTC	
DMI2-F	TCGAGGTACCATGATGGAGTTACAAGTTATTAGG	DMI2-HA sequencing
DMI2-R	CTACGTCGACTCTCGGTTGAGGGTGTGACAAGGT	
DMI2-F1	AGAACAGTTACACTGCCTTG	
DMI2-F2	GAGGAACTGCAGGGTATCTG	
MtCERK1-F	TCGAGGTACCATGGAACATCAACCCAGATTCACCT	MtCERK1-HA sequencing
MtCERK1-R	CAACGTCGACTCTTCCTGACATAAGATTCACAAGG	
MtCERK1-F1	ATCAAGGGAGTGGCATTGTTTTTG	
