# Supplementary material for: A combination of chitooligosaccharide and lipochitooligosaccharide recognition promotes arbuscular mycorrhizal associations in Medicago truncatula
Source: Nat Commun. 2019 Nov 6;10:5047. doi: 10.1038/s41467-019-12999-5 (PMC6834629; doi:10.1038/s41467-019-12999-5)
Supplement: Supplementary file 3 — Description of Additional Supplementary Files [file 41467_2019_12999_MOESM3_ESM.pdf]

Description of Additional Supplementary Files

File Name: Supplementary Data 1
Description: Genes regulated by COs, flg22 and SmLCO in M. truncatula wild type and receptor mutants. Related to Fig. 4a. Gene expression levels are shown in the table with log2 fold change and FDR correction.
